# Ionic liquids adsorption and interfacial tension reduction for synthetic resinous and asphaltenic oils: salinity and pH effects

**DOI:** 10.1038/s41598-024-59472-y

**Published:** 2024-04-24

**Authors:** Seyed Ehsan Moradi, Seyednooroldin Hosseini, Naser Akhlaghi, Mostafa Narimani, Elias Ghaleh Golab

**Affiliations:** 1 Department of Chemical Engineering, Omidiyeh Branch, Islamic Azad University, Omidiyeh, Iran; 2 Department of Petroleum Engineering, EOR Research Center, Omidiyeh Branch, Islamic Azad University, Box Post: 164, Omidiyeh, 63731-93719 Khuzestan Iran

**Keywords:** Saponification, Alkaline flooding, Resin, Asphaltene, pH, Ionic liquid, Chemistry, Energy science and technology, Engineering, Materials science

## Abstract

The effects of sulfate salts under low and high salinity conditions and pH of 3.5–11 on interfacial tension (IFT) reduction and IL adsorption using resinous (RSO) and asphaltenic (8 wt/wt%) synthetic oils are investigated. The measurements showed the increasing effect of pH on the IFT of RSO/DW from 23.5 to 27.3 mN/m (pH = 3.5 → 7) in the first place and a reducing effect (0.4 mN/m) if pH = 7 → 11. Using a high concentration of 50,000 ppm for MgSO_4_, and Na_2_SO_4_ revealed an extensive IFT reduction for a pH value of 11 with the value of 0.20 mN/m for Na_2_SO_4_. The measured IFT values showed the significant impact of IL (500 ppm) on the IFT (minimum value of 0.01 mN/m for RSO/50,000 Na_2_SO_4_ + 500 ppm 1-decyl-3-methyl imidazolium triflate ([C_10_mim][TfO])) for pH = 11. The IL adsorption measurements showed the role of in-situ surfactant production (saponification process) on the 1-decyl-3-methyl imidazolium chloride ([C_10_mim][Cl]) and [C_10_mim][TfO] adsorption reduction from 3.67 to 2.33 and 4.21 to 3.34 mg IL/g rock, respectively. The performed core flooding experiments using the optimum chemical formulation showed the possibility of tertiary oil recovery with maximum oil recovery of 28.8% based on original oil in place in the presence of 500 ppm.

## Introduction

Among the chemical enhanced oil recovery (CEOR) injection of the alkaline solution is highly effective, especially as a relatively inexpensive chemical method for higher oil production using pH manipulation^1,2^. The appearance of such a method dates back to 1917 when F. Squires proposed this method for oil displacement. Unfortunately, although this method is old enough to be utilized on the field scale, only a few industrial efforts can be found performed with success. Fortunately, regarding the progress achieved through chemical synthesizing, alkaline flooding gained increasing attention due to its cost and relative simplicity. Injection of alkali is effective for oil recovery since it can produce in-situ soap and surfactant for interfacial tension (IFT) reduction purposes as the alkali reacts with the acidic content of the crude oil. Also, there are other mechanisms such as entrainment and entrapment along with emulsification^3–5^, changing wettability from oil-wet to water-wet or water-wet to oil-wet^6–8^, coalescence and emulsification^9^, and mobility control by injecting the caustic solution during the alkaline flooding process. A detailed description about the aforementioned mechanism are discussed by Johnson^10^ during 1976 with a main focus on mechanisms by which caustic waterflooding may improve oil recovery. As alkaline reacts with rock in the presence of divalent ions, precipitation can occur preferentially reducing high-permeability channels and then affecting the sweep efficiency^11^ known as mobility-controlled caustic flood (MCCF). Among these possible mechanisms, IFT reduction is essential since it has a direct impact on the capillary number and also reduces the residual oil saturation to zero if a proper capillary number is achieved. In this way, the investigations showed that as the capillary number changes, an increase in the efficiency of microscopic displacement is inevitable^12,13^. The other point is that increasing the capillary number to a desired value is capable of reducing the hysteresis of imbibition-drainage and residual oil saturation (ROS)^14,15^.

The point is that the alkali injection and in-situ soap production are highly dependent on the salt concentrations and pH value besides the resin and asphaltene contents of crude oils. So, it is highly vital to perform investigations to see the possible synergy or antagonistic relation between these parameters. The crude oil composition is highly important since the naphthenic acid fraction is a mixture of several hydrocarbons of carboxy phenols and cyclohexyl carboxylic acids^16,17^, etc^18,19^. which each of these fractions has its impact on the saponification process.

In this way, some limited research are performed such as the investigation performed by Buckley and Fan^20^ considering the pH, asphaltene fraction quantity and acid number, they reported that the impact of chemical surfactants is completely different from the impact of asphaltene fraction as natural surfactant on the IFT reduction.

In other words, although the impact of conventional chemical surfactants on the IFT reduction and oil recovery was investigated through the literature, there is a limited number of investigations for the effect of indigenous surfactants (i.e. resin and asphaltene) on the IFT reduction and their probable relation to the other operating of pH.and thermodynamic parameters. For example, Almalik et al.^21^ examined the effect of NaOH as an inexpensive alkali on oil recovery using the IFT reduction mechanism and synthetic crude oils prepared with isolated resin and asphaltene fractions.

Besides the impacts of pH and the crude oil type, the studies revealed that the effect of salts and salinity is an undeniable parameter that must be carefully examined in the absence and presence of chemicals. In this way, several studies have performed IFT measurements using wide ranges of salt concentrations and types besides the thermodynamic conditions to find the possible synergy between different chemicals and crude oil composition. So, it is highly required to design and perform investigations that take into account the effects of ions and oil compositions as the IFT is set as an objective function.

On the other side, it is well established that using surfactants can reduce water/oil interfacial tension (IFT) to a level that can form an oil bank using the trapped oil and mobilize the oil bank toward the production point. Unfortunately, since the surfactants are mostly toxic, costly, and faced with low functionality and even degradation under harsh salinity and temperature conditions, it is highly required to investigate the interactions between surfactants and different operating conditions to find the best choice for EOR purposes^22^. The other limitation that the petroleum men faced during the application of surfactants in enhanced oil recovery (EOR) processes is retention of surfactant which directly affects the economics of the EOR process^23^. In detail, besides the stable structure under harsh salinity or temperature conditions, surfactant retention and adsorption are highly important to consider whether one surfactant is a cost-effective chemical or not^23^. In detail, surfactant injection for higher oil production generally suffers the drawback of a significant surfactant loss due to retention in the porous media^24–26^.

Generally, although complete surfactant adsorption is impossible, it is possible to overcome surfactant precipitation, and phase trapping using a proper surfactant that can tolerate harsh salinity and temperature. So it is only possible to control and reduce the adsorption amount to a minimum value^27,28^. For this purpose, it is required to measure the surfactant adsorption under different conditions such as different salt concentrations, pH, and rock types to find a way which can help to control the surfactant adsorption. Reducing the surfactant adsorption is important since it directly affects the effectiveness and the cost of the process since in the usual cases, half of the chemical injection project cost is related to the amount of used surfactant^24,27–30^.

Therefore, it is vital to minimize surfactant adsorption as a key factor in achieving and designing an economic surfactant flooding process^31^. In detail, the surfactant adsorption on the rock surface or partitioning into crude oil can directly affect the efficiency of the surfactant flooding process since any reduction in the surfactant concentration influences the surfactant functionality for IFT reduction^32,33^. Respecting the importance of this concern, a comprehensive review was performed by Belhaj et al.^34^ to describe the effect of different parameters namely salinity, surfactant type, crude oil composition, etc. on surfactant adsorption.

In light of these limitations, the researchers proposed several solutions to overcome such limitations such as using a combination of surfactants with other chemicals such as alkali and polymer as the common combinations. As previously mentioned, alkalis are responsible for further IFT reduction in the presence of conventional surfactants under the saponification process leading to the formation of in-situ surfactants which is the consequence of alkali and the naphthenic acids reactions^35,36^.

The point is that the produced in-situ surfactants are effective on the tertiary oil recovery not only by IFT reduction but also by sacrificing themselves to be adsorbed on the rock surface before the injected surfactant^29,37^. In total one can conclude that not only the IFT reduces in the first place the pH can be enhanced in the aqueous phase and reduce the usage of surfactant and the related operating cost^29^.

Besides the effectiveness of the in-situ surfactant for surfactant adsorption prevention, the researchers have proposed a new class of surfactants namely ionic liquids (ILs) which can tolerate very high salt concentrations and even high-temperature conditions^38^. In general, ILs are known as salts with melting or glass transition temperatures below 100 °C which is low enough that put most of the ILs at the liquids phase under room temperature, which means easy handling and application of these chemicals. Moreover, since they have negligible vapor pressure concomitant with high thermal stability (at temperature less than or equal to 100 °C) and solvating capacity, they are a good candidate for EOR purposes^39^, Hezave et al.^40–43^. The other interesting advantage of the ILs which put them at the top of the list of researchers is their tunability. In detail, since it is possible to use different cations and anions to produce and fabricate different ILs, producing any specific task IL for any candidate reservoir is feasible^44,45^. In addition, there are investigations performed to find the impact of different types of ILs and functional groups on IFT reduction similar to the investigation performed by Najimi et al.^43^. Besides the unique features of the ILs, the high thermal stability and low volatility of ILs lead to environmental issues such as water pollution. In detail, although, the low vapor pressure of ILs reduces the risk of air pollution, calling these solvents “green” solvents is far from the reality which must be considered as their intrinsic risk as they are going to be used in industries especially those are connected to water sources and aquifer such as EOR processes. In other words, although the application of ILs has several advantages, it is highly required to analyze these ILs for environmental issues to find more realistic solutions. In this way, it is necessary to perform different independent investigations to have good insights into the advantages and disadvantages of using ILs as a new class of solvents. For example, due to the high stability of ILs in water, these compounds could become persistent pollutants in wastewater. For this reason, it is a priority to determine the further consequences and the environmental risk of the presence of ILs in wastewater. In this way, Zabihi et al.^46^ examined conventional (sodium dodecylbenzene sulfonate (SDBS)) and IL-based (imidazolium and pyridinium chlorides) surfactants as two different classes of surfactants. They reported that both of the examined surfactants were capable of tolerating harsh salinity conditions up to 50,000 ppm although using SDBS concentration higher than 1600 ppm led to fast precipitation in the aqueous solution while the examined 1-dodecyl 3-methyl imidazolium chloride ([C_12_mim][Cl]) and 1-octadecyl 3-methyl imidazolium chloride ([C_18_mim][Cl]) showed the best IFT reduction capability without any precipitation. Furthermore, Somoza et al.^47^ investigated the effect of 1-decyl-3-methyl imidazolium triflate ([C_10_mim][OTf]) on the IFT of crude oil/aqueous solution. They reported that increasing the concentration of IL in the range of 2000, 3000, 4000, and 5000 ppm led to IFT reduction to values of 6.70, 2.42, 0.36, and 0.41 mN/m, respectively, using distilled water (DW). They also reported that as the concentration of NaCl was increased from 0.1 to 1 wt%, the IFT value would increase from 0.36 to 0.57 mN/m and 0.93 mN/m while further increase in the NaCl concentration to values of 2 and 4 wt% had no significant effect on the IFT regardless of increasing or decreasing trend.

So, the effects of sulfate salts sodium sulfate (Na_2_SO_4_), magnesium sulfate (MgSO_4_), and calcium sulfate (CaSO_4_) in the low salinity range concentrations of 1500, 5000, and 5000 ppm, respectively) and high salinity conditions of 50,000 ppm for both MgSO_4_ and Na_2_SO_4_ as one of the most important salts were investigated on the IFT reduction for the first time. Besides, the impact of acidic, basic, and neutral conditions was carefully examined in the presence and absence of 1-decyl-3-methyl imidazolium chloride ([C_10_mim][Cl]) and 1-decyl-3-methylimidazolium triflate ([C_10_mim][OTf]) on IFT and IL adsorption as a new class of surfactants considering the possible saponification process may occur due to presence of alkali and acidic contents of the oil sample.

Besides, to enhance the applicability of the obtained results, synthetic crude oils with resin and asphaltene fractions were prepared and used as the sample oil using the IP143/90 method to isolate the required resin and asphaltene fractions for sample oil preparation. Using these synthetic oils is highly effective in finding a more generalized relation between the chemicals, pH, and crude oil since these two fractions are among the most effective fractions of each crude oil. Besides, the current work is concentrated on examining the role of different possible mechanisms on tertiary oil recovery using several core flooding experiments with the optimum chemical formation.

The worth mentioning point is that the current investigation was performed since there is no report on the impact of two different ILs from imidazolium and triflate families using resinous and asphaltenic synthetic oils. In detail, the majority of EOR investigations focused on the application of crude oil from different oil fields while the application of resin and asphaltene fractions to prepare the synthetic oils are very limited in number. Besides, since the surface activity of the resin and asphaltene is well established, it is crucial to investigate the interactions between different chemicals and these two active fractions which are so complex in structure.

Moreover, there are a very limited number of investigations regarding the adsorption of the ILs from two families compared with each other under different pH values using synthetic resinous and asphaltenic oils. In detail, using synthetic oils and different families of ILs as the main core of investigation provides better insight into the possible interactions and synergies between these two chemicals. Furthermore, there is a limited number of investigations regarding the impact of sulfate salts on the IFT especially with the main focus on the application of synthetic resinous and asphaltenic oils.

## Material and method

### Material and solutions

The used sample crude oil for resin and asphaltene isolation was supplied from Offshore Oil Company, Iran. The analysis showed that the used crude oil comprised of 7.1% and 9.8% asphaltene and resin and the fractions of other compartments were N. Paraffins (12.7%), I. Paraffins (10.5%), Olefinic (0.1%), Naphthenes (12.1%), Aromatics (5.7%), Saturates (C_15_ < C_20_) (5.2%), Aromatics (C_15_ < C < C_20_) (4.3%), Unknowns (C < 20) (13.3%), C_20_^+^ (19.2%).

Besides, the required chemicals and salts were supplied with a minimum of 99.5% from Merck, USA (calcium sulfate, sodium sulfate, magnesium sulfate). the salts were used to prepare the aqueous solutions with a concentration of 5000 ppm and 50,000 for Na_2_SO_4_, and MgSO_4_ except for CaSO_4_ since the maximum solubility of CaSO_4_ was 1500 ppm without no precipitation. In detail, CaSO_4_, concentration was selected with values lower than 5000 pm since only for concentrations below 2100 ppm no precipitation occurs for this salt. So, CaSO_4_ with a concentration of 1500 ppm was prepared which is well below the precipitation threshold of this salt. Moreover, [C_10_mim][OTf] (CAS number of 412009-62-2) with a purity of 98% was supplied from Nanjing Pars Biochem Co., Ltd, China, while the required [C_10_mim][Cl] (CAS number of 171058-18-7) was supplied from Merck, KGaA, Darmstadt, Germany with purity better than 95% (see Table 1).

**Table 1:** The properties of the used IL.

No	Name	Molecular structure	Chemical formula	CAS number	Molecular weight
1	1-Decyl-3-methylimidazolium chloride		C_14_H_27_ClN_2_	171058-18-7	258.83
2	1-Decyl-3-methylimidazolium triflate		C_15_H_27_F_3_N_2_O_3_S	412009-62-2	372.447

### Extraction of asphaltene and resin

Since crude oil is a combination of a large number of different types of compounds, using crude oil as the sample oil brings some ambiguities into the obtained results and provides some unreliability during the interpretation of the obtained results. In this way, one of the solutions to resolve these ambiguities is using synthetic oils prepared from one specific fraction of crude oils such as resin and asphaltene fractions. In other words, although crude oil is comprised of four fractions of resin, asphaltene saturates and aromatics, these resin and asphaltene fractions have a considerable impact on the interactions between chemicals, salts, pH, and other parameters since they can act as a natural surfactant. So, instead of using only one molecular weight and composition for crude oil which is impractical^48^, it is more applicable to use hydrocarbon group type analysis^49,50^. In light of these facts, asphaltene and resin fractions which are natural surfactants can manipulate the IFT values and stability of crude oil/water emulsion due to their structure and heteroatoms that exist in their molecular structure^51^ although the information regarding the resin fraction is lower than the asphaltene fraction^52,53^.

In general, one of the criteria that can be used to characterize the resin and asphaltene fractions is the H/C ratio which is between 1.2 and 1.7 for resin fraction and between 0.9 and 1.2 for asphaltene fraction. Generally, although resin and asphaltene are similar to each other, resins are smaller than asphaltene molecules with a more branched structure with a higher content of naphthenic acids^54^.

In this way, the IP 143/90 approach was used to extract the fractions of the asphaltene and resin from crude oil, and then they were dissolved in toluene to prepare resinous and asphaltenic synthetic oils for the investigations. The point is that although resin and asphaltene fractions are essential due to their complex structure, surface activity nature, etc., their role in the IFT reduction and wettability alteration under different operating conditions especially pH value is limitedly examined^55^ in particular for resin fractions which mostly acts as the asphaltene stabilizer chemical. The reason behind the surface activity of these two fractions proven by spectroscopic is that these two fractions are mostly compromised of hydroxyl groups, ester, acid, carbonyl functions, and long paraffinic chains with naphthenic rings and polar functions present in the structure^56,57^. The point besides this analysis is that although these two fractions are similar to each other considering the chemical groups, they are different in aromaticity, size polarity, and even physical appearance^58^. In brief, the asphaltene fraction was isolated using n-heptane (40:1 ratio) and then further purified with soxhlet recycling. After that, the remaining de-asphalted residue was used to isolate the resin fraction using the column chromatography method described in detail elsewhere^59,60^. In brief, the de-asphalted oil + n-heptane which is a molten-like residue was contacted with a silica gel column with a mesh of 35 − 70 mesh according to the required ASTM to be adsorbed. Then the resultant mixture was rinsed with a mixture of 70:30 n-heptane and toluene to lose saturates and aromatics. Finally, a ternary mixture of acetone/dichloromethane/toluene with a ratio of 40:30:30 was used to extract the resins from the column^61–63^ (see Table 2).

**Table 2:** Elemental analysis of heteroatoms of resin and asphlatene fractions.

H/C ratio	wt % of heteroatoms of resin and asphaltene fractions				Fractions	Total acid number
	S, O and N	S	O	N		
1.12	11.01	3.71	7.30	0.00	Asphaltene	0.56
1.44	10.35	6.59	3.76	0.00	Resin	0.67

A glance into the tabulated results in Table 2 shows that the asphaltene fraction is less branched than the resin fraction since the asphaltene fraction has the H/C ratio or aromaticity index of 1.12 while the resin fraction has the H/C ratio or aromaticity of about 1.44. As a consequence of this more branched structure of resin than asphaltene fraction, it can introduce more surface activity than asphaltene. In this way, resin fraction is more capable of having interactions with the other charged particles, surfaces, etc. for more effective impact during the IFT reduction and wettability alteration purposes. In general, both asphaltene and resin fractions comprised of heteroatoms of sulfur and oxygen with negative charges make the resin molecules similar to surfactant molecules comprised of head and tail.

### IFT measurement

Using the pendant drop method for rising or pendant drop at the tip of the nozzle is increasing since it is a highly reliable and accurate method for IFT measurement up to 0.1 mN/m or somehow lower values^63^ (Fanavari Atiyeh Pouyandegan Exir Co., (APEX Technologies co.,), Arak, Iran).

Different sections of the equipment that can be used to measure the IFT with this technique are online image-processing software, a dosing system with moving stages, and an image-capturing system. The dosing system is responsible for injecting the required volume of a drop at a desired volume in a way that the drop is suspended at the tip of the nozzle for the shape analysis stage. Depending on the used nozzle size, it is possible to measure IFT values which means different volumes of liquid must be injected and suspended. The point is that the shape of the nozzle (straight or U-type) is selected according to the difference between the density difference between the bulk and drop phase. If the density difference between the drop and bulk is negative, the U-shape nozzle must be used since the drop would be in a rising position. But if the density difference between the drop and bulk phase is positive, the straight nozzle must be used since the drop would be in a pendant (downward) position. The next section is the image capturing section which is comprised of a macro lens and a CCD camera with proper resolution which can be used to capture and dispatch the required images to the online software (Apex technologies Co. drop shape analysis software.V.2023). The capturing system is selected and installed in a way that the operator is capable of magnifying the formed drop to occupy the software screen as much as it is possible. The reason is that the last section (software section) utilizes equations and shape factors that are highly dependent on the magnification of the drop. In other words, higher magnification means a higher possibility for the software to determine the borders and shape of the drop for more accurate conversion of the measured parameters to IFT values using the following Eq. (1):

1 $$\gamma =\frac{\Delta \rho g{ D}^{2}}{H}$$

where, *Δρ* is the density difference for drop (oil drop) and bulk (aqueous solution), *g* is the acceleration of gravity, and *H* is the shape-dependent parameter which depends on the shape factor value, i.e. *S* = *d/D*, where *D* is the equatorial diameter and *d* is the diameter at the distance *D* from the top of the drop^64–66^.

The point that must be mentioned is that during the pendant drop IFT measurement stage, each reported data point is the average of at least three independent measurements with a maximum uncertainty of ± 0.2 mN/m1 which was impossible to be indicated in the plotted IFT values due to a broad range of measured IFT values.

In the next stage of this section, the IFT values for the systems with IFT values lower than 0.5 mN/m, the spinning drop method can be used. This method which was first developed by Vonnegut^67^ is mainly based on the drop deformation and length variation due to centrifugal force. In detail, as the drop was rotated at a high speed not more than 3000 rpm, it tended to reshape and lose its drop (spherical) shape into a cylinder. At this point and if the L/D passes 4, the IFT value can be calculated using the following Eq. 2:

2 $$\gamma \, = \, \left( {\Delta \rho \omega^{{2}} {\text{R}}^{{3}} } \right)/{4}$$

where the ∆ρ and ω are the bulk and drop phase densities difference (Kg/m^3^), and the angular velocity (rad/s). In practice, better results can be archived if the L/D value well exceeds 4 to reach a real cylinder shape.

In brief, 2 cc of the bulk phase was first injected into the transparent chamber. After that, a 10-µl syringe was used to inject a drop with the volume of a few microliters middle of the bulk phase as the system is rotating with a low angular velocity. This low angular velocity at the beginning stage of drop injection is required to prevent the adhesion of the oil drop at the glass surface. As the drop was placed in the bulk phase, the velocity was gradually increased to a point that the L/D for the drop was achieved and passed. At this point, the IFT can be measured using the aforementioned equation.

### Core flooding experiment (forced imbibition test)

**Figure 1:**
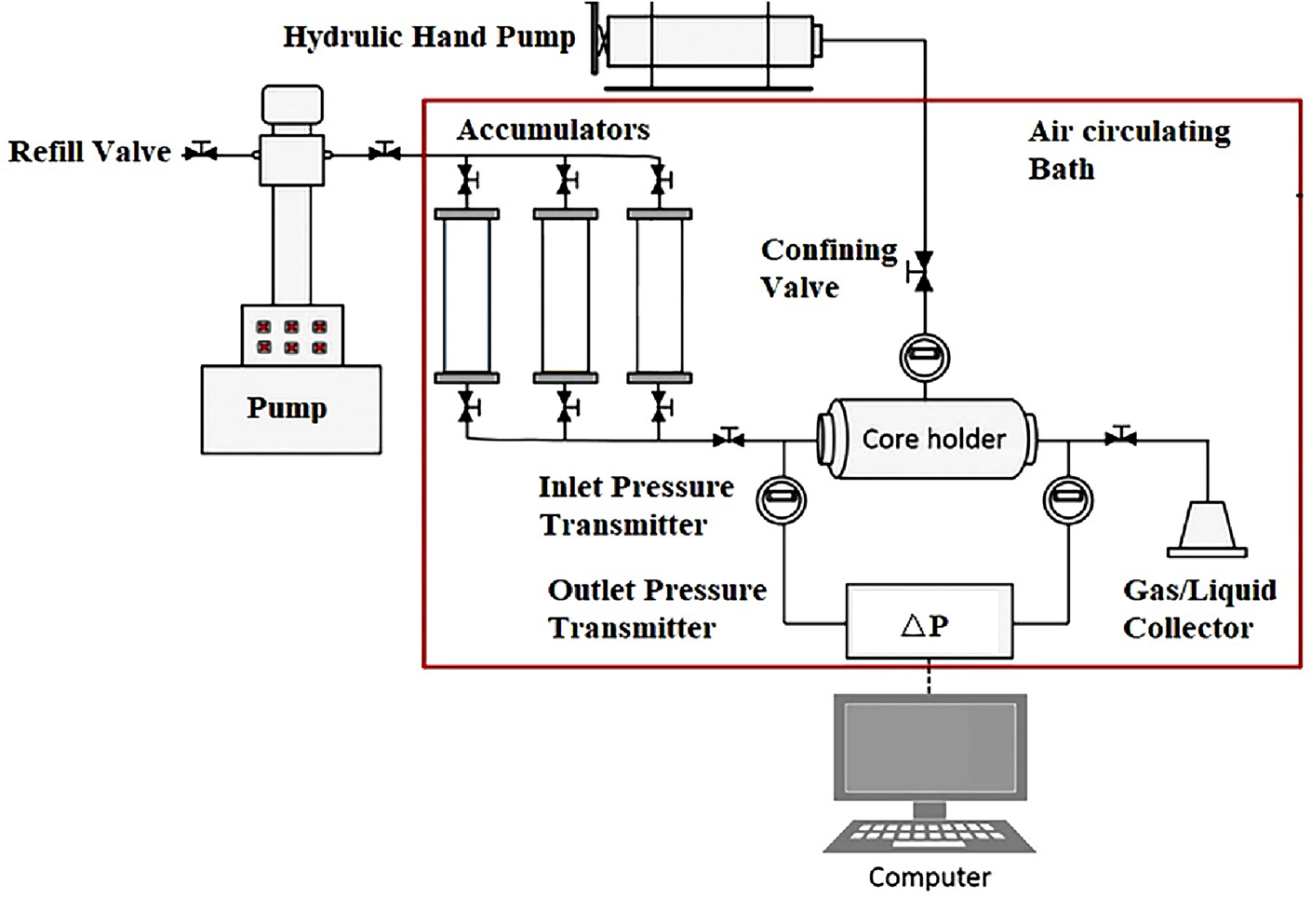
The schematic of core flooding apparatus.

A core flooding apparatus designed for temperature and pressure up to 150 °C and 700 bar, respectively was used to perform the required core flooding experiments (Fanavari Atiyeh Pouyandegan Exir Co., (APEX Technologies co.,), Arak, Iran) (see Fig. 1).

The core flooding equipment is equipped with a high-pressure fluid injection pump which can inject the desired fluids in the range of 0.001–15 cc/min with a maximum pressure of 600 bar with different modes of constant injection flow rate and constant pressure. In brief, the desired fluids including synthetic oil, chemical slug, or formation brine can be kept ready for injection using three fluid accumulators or transfer vessels rated for pressure up to 700 bar.

The system is also equipped with a hassler core holder to handle the core with a different length between 5 and 12 cm with dimeter of 3.8 mm. The confining pressure which surrounds the core was applied to the core using a high-pressure hand pump. The point is that the required confining pressure was applied around the core only 50 bar higher than the pore pressure to avoid core collapse due to high confining pressure which can destroy the structure of the core. The core flooding apparatus was also equipped with a data acquisition system to record the temperature of the system, inlet and outlet pressures, and parameters of the high-pressure pump. Moreover, a gas back pressure regulator (GBPR) was installed in the outlet section of the core holder. This section is required to maintain the inside oppressor of the core holder at a desired pressure if the operator wants to mimic the reservoir pressure into the core. Considering the above points and the required injection sequence, the following procedure can be used to perform the core flooding experiment:

- Placing the core with known porosity and permeability inside the core holder
- Formation brine injection with several pore volumes (PVs) to ensure core saturation with formation brine
- Injection of synthetic oil to the saturated core to a point no water was produced (irreducible water saturation (S_wir_))
- Injection of formation brine to mimic the secondary oil recovery to a point that no oil was produced (residual oil saturation (S_ro_))
- Optimum chemical formulation slug injection with 0.3 PV and injection flow rate of 0.3 cc (the fluid movement in the reservoir is in laminar regime with flow rate of 1 ft/day which is similar to cc/min)

The point is that in the last stage of this procedure, it is possible to investigate the effect of operating parameters such as the slug size and injection flow rate if they are selected as the objective function of the study which is not selected in the current investigation.

### Total acid/base number (TAN/TBN)

The sample crude oils from two different oil reservoirs were used to extract the required resin and asphaltene fractions for preparing the resinous synthetic oil (RSO) and asphaletenic synthetic oil (ASO). The total acid number (TAN) of the used crude oils was measured since it can show the crude oil acidity or neutrality. In detail, crude oil is acidic type if its TAN number is higher than 0.5 mg KOH/g oil^55^. The determined acid number is often considerably affected by carboxyl groups that exist in asphaltenes and resins The TAN of the crude oils was measured using a potentiometric titration based on the ASTM D 664 method. The RSO sample had a high acid fraction (TAN = 1.81 mg KOH/g oil), and the ASO sample had a low acid fraction (TAN = 0.45 mg KOH/ g oil).

### Dynamic adsorption test

In the current investigation, the adsorption of the IL as a surfactant was examined via a dynamic adsorption procedure as previously performed by Hezave et al.^40^ under room temperature.

In detail, in the first place, the required chemical solutions were prepared with known compositions, and the values were recorded. After that, several PVs of each chemical formulation (maximum PVs of 12) with known concentration were injected into the selected cores using the core flooding apparatus. The core flooding system was rated for pressure and temperature of 600 bar and 150 °C with three different accumulators and one Hassler-type core holder.

For each adsorption test, the concentration of IL was set at 1500 ppm (above the critical micelle concentration (CMC) to ensure achieving the ultimate effect of IL in the system). After preparing the solution, the aqueous solution was injected with a flow rate of 0.3 cc/min similar to the flow rate of fluids in the oil reservoir (1 ft/day) into the cores. In this way, the core was flooded with several PVs of aqueous solution to reach full saturation with formation brine. After that, the saturated core with formation brine was flooded using selected crude oils to reach a point of no aqueous solution was produced which means the core is at the irreducible water saturation. During the injection of the aqueous solution, the effluent fractions were collected and analyzed using UV spectrophotometry (Thermo Scientific GENESYS 10 UV Visible Spectrophotometer, USA). Since the surfactant has a maximum absorbance at 212 nm, the effluents were well diluted to ensure reaching a concentration that the linear calibration curve is valid.

## Result and discussion

### Evaluation of performance of pH variation on the IFT of oleic phase under low salinity conditions

**Figure 2:**
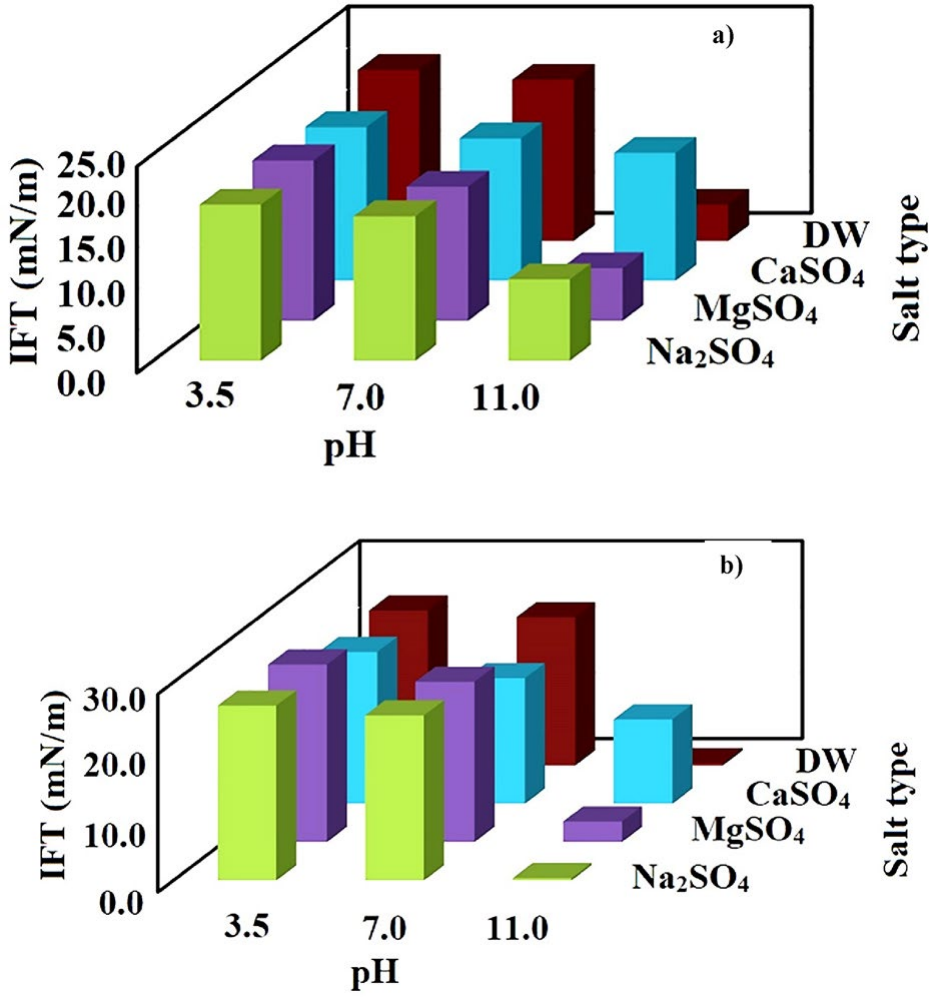
IFT values of (**a**) asphaltenic model oil/brines at different pH conditions, (**b**) resinous model oil/brines at different pH values and different sulfate salts.

In the first place, the effects of CaSO_4_ (1000 ppm), MgSO_4_ (5000 ppm), and Na_2_SO_4_ (5000 ppm) at different pH values of 3.5, 7, and 11 were studied on IFT variation of asphaltenic and resinous synthetic oils (see Fig. 2). The point is that since the IFT measurement needs the density of different phases, the densities of different solutions were measured in the first stage of this phase (Table 3) using gravimetric and volumetric methods including glass pycnometer and analytical balance (max weighing capacity = 120 g with accuracy of 0.1 mg, Sartorius, Germany).

**Table 3:** The measured density values.

Concentration (ppm)	Na_2_SO_4_	MgSO_4_	CaSO_4_
0	0.9979	0.9979	0.9979
1000	0.9986	0.9995	0.9980
5000	1.0021	1.0041	–
50,000	1.037	1.052	–

The results revealed that, for the case of DW, increasing pH from 3.5 to 11 has a significant impact on the IFT reduction since the saponification which is responsible for in-situ surfactant production occur (see Fig. 2b) (see Table 4). The saponification occurs between an organic acid and alkali to form soap using the following reaction:

1 $${\text{HA}} + {\text{OH}}^{ - } \leftrightarrow {\text{A}}^{ - } + {\text{ H}}_{{2}} {\text{O}}$$

**Table 4:** The measured IFT values under different pH and low salinity condition.

	IFT (mN/m)					
	pH = 3.5		pH = 7		pH = 11	
	RSO	ASO	RSO	ASO	RSO	ASO
Distilled water (DW)	23.5	20.7	27.3	19.5	0.4	4.4
CaSO_4_	23.0	18.6	2.3	3.4	13.5	18.3
MgSO_4_	26.9	19.4	3.3	6.0	3.1	6.4
Na_2_SO_4_	26.5	18.9	2.0	3.3	0.4	9.9

As this reaction occurs between the naphtenic acid parts of the RSO and ASO and the caustic alkali, in-situ soap generation is inevitable leading to lower IFT values in the primary stages.

In the next phase of the experiments, the impact of sulfate salts which is the most effective salts in the EOR industries was investigated on the IFT reduction.

A glance into the results depicted in Fig. 2b demonstrated that CaSO_4_ and MgSO_4_ reduce the efficiency of increasing pH on the IFT values compared with the IFT values reported for DW, although for the case of CaSO_4_, this reduction is more evident. The reason behind this observed trend can be related to the larger radius of calcium than magnesium can adsorb more produced soap molecules in the solution and form complex molecules preventing the accumulation and packing of those surfactant-like molecules at the interface leading to larger IFT values for the solutions including CaSO_4_ than MgSO_4_ or Na_2_SO_4_. Similarly, between solutions comprising MgSO_4_ and Na_2_SO_4_, the IFT value of the solution with Na_2_SO_4_ is lower than the solution prepared by MgSO_4_. This observed trend correlated to the effect of Na^+^ on the resin fraction of the examined acidic crude oil. As aforementioned, the crude oil comprises resin fraction that can act as a natural surfactant. These natural surfactants can reduce the IFT value if well-packed at the interface.

Unfortunately, resin molecules are large molecules (empirical formulation of C_60_H_87_O_4_S according to the elemental analysis aforementioned in the section under the heading of “*Extraction of Asphaltene and Resin*”) that need to pack far from each other for more stabilized conditions. So, the accumulation and packing of these molecules at the interface are limited due to their size, and surface charges introduce repulsive force if they come into the interface. On the other hand, if Na^+^ cation interacts with negative heads comprised of heteroatoms of sulfur or oxygen, the surface charge reduces, providing more chances for resin molecules to reach the interface and reducing the IFT value.

In the next series of experiments, the impact of asphaltene fraction was examined on the IFT reduction compared with the resinous synthetic oil (see Fig. 2a). The results revealed that the IFT reduction in asphaltenic solution is lower than the resinous synthetic oil (Fig. 2b). This trend contributed to the lower acidic contents of asphaltenic synthetic oil (TAN = 0.45) than resinous synthetic oil (TAN = 1.81), and crude oil (TAN = 1.41) means the lower capability of producing in-situ soaps and IFT reduction. The point is that the resinous synthetic oil and Na_2_SO_4_ have lower IFT values than the IFT value obtained for the crude oil measured. So, it seems the existence of other fractions can act as a barrier for resin fraction for IFT reduction.

### Evaluation of performance of pH variation on the IFT of oleic phase under high salinity conditions

**Figure 3:**
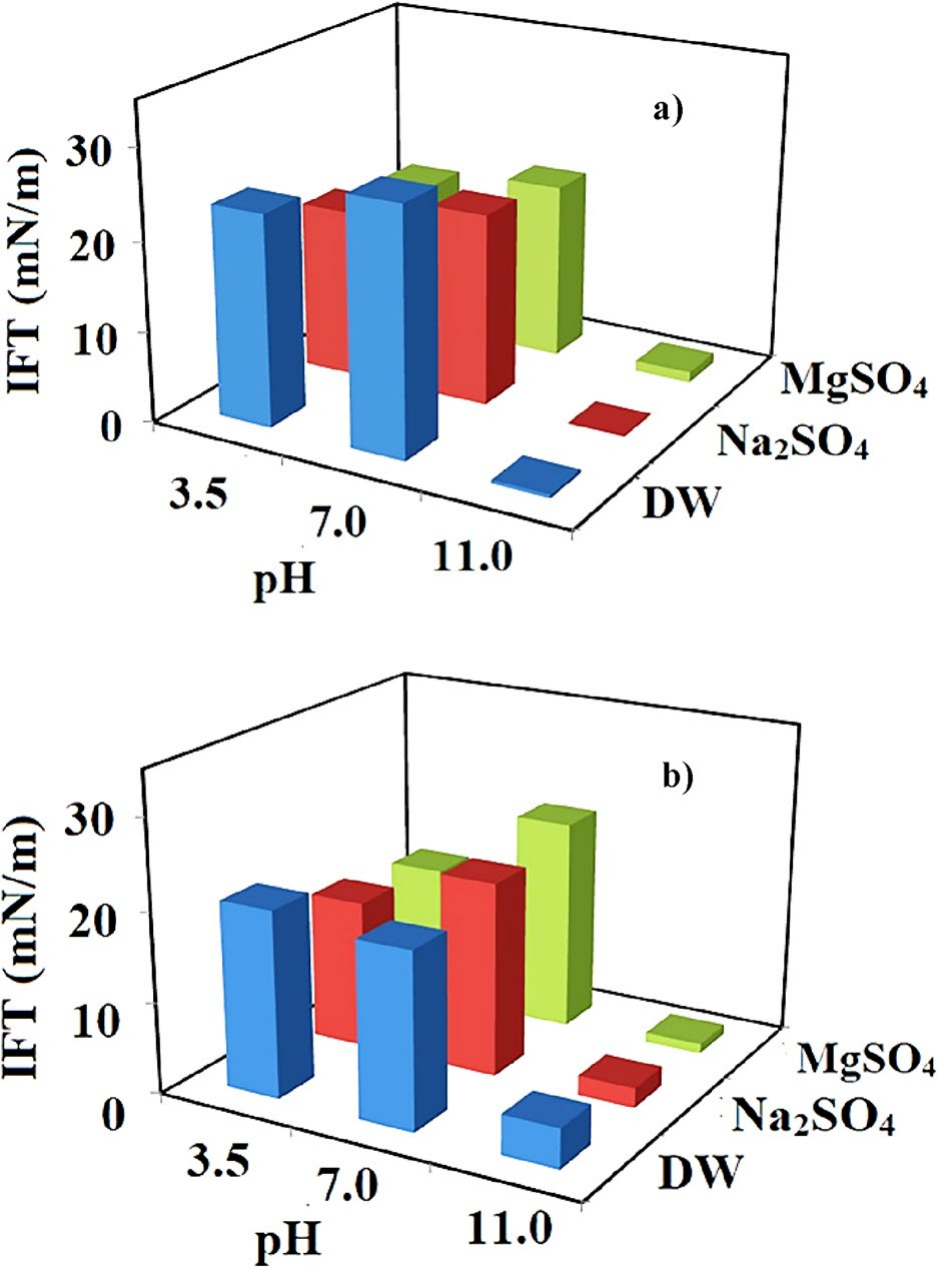
IFT values of (**a**) resinous model oil /brines for different pH conditions, (**b**) asphaltenic model oil /brines for different pH conditions at the presence of different sulfate salts.

In the current section, the effect of high salinity conditions for Na_2_SO_4_ and MgSO_4_ under a concentration of 50,000 ppm was investigated on the IFT of different sample oils of asphaltenic, and resinous synthetic oils. The obtained results tabulated in Fig. 3 (see Table 5) demonstrated that the impact of pH for IFT reduction moved to MgSO_4_ instead of Na_2_SO_4_ for RSO while a shifting point at a pH value of 7 can be observed for both examined synthetic oil and all of the examined aqueous solutions of DW, 50,000 ppm of Na_2_SO_4_ and MgSO_4_.

**Table 5:** The measured IFT values under different pH and high salinity condition.

	IFT (mN/m)					
	pH = 3.5		pH = 7		pH = 11	
	RSO	ASO	RSO	ASO	RSO	ASO
Distilled water (DW)	23.5	20.7	27.3	19.5	0.4	4.4
MgSO_4_	19.6	16.2	21.1	21.6	1.10	0.9
Na_2_SO_4_	20.7	16.7	21.7	19.7	0.10	2.3

The results revealed that tat in contrast to the results obtained for RSO under low salinity conditions and a pH value of 3.5, the presence of salts under high salinity conditions led to lower IFT values at a pH value of 3.5 with a minimum value of 17.3 mN/m for MgSO_4_.

The reason behind this obtained trend can be correlated to the synergy between the high concentration of ions and produced H^+^ besides the chemical structure of resin fraction has more branches giving it a surface activity that can be accumulated in the interface for IFT reduction purposes. In detail, the surface activity of the resin molecules along with the high concentration of ions that can be trapped in the molecular structure of the complex resin molecules can reduce the repulsive forces that may exist between the resin molecules even if the aqueous solution is in its acidic condition (pH value of 3.5) consequently leading to accumulation of a higher number of resin molecules in the interface and IFT reduction and reached to its ultimate effect for pH value of 11 that in-situ soap production boost this phenomenon leading to an extensive reduction in IFT value (minimum value of 0.1 for Na_2_SO_4_).

A closer look into the results depicted in Fig. 3 revealed that although for the low salinity conditions, the presence of salts led to IFT reduction for all of the examined salts in the presence of ASO, the reducing trend for the pH value of 7 changed to increasing trend (for high salinity condition) while for the pH values of 3.5 and 11 the reducing trend remained unchanged and even with higher impact. The interesting point is that similar to the other examined systems, the pH value of 7 is a shifting point where the IFT increases as the pH value increases from 3.5 to 7 while a further increase in the pH value from 7 to 11 leads to extensive IFT reduction. According to these findings, it seems that using the Na_2_SO_4_ and MgSO_4_ salts under a high salinity condition of 50,000 ppm and pH value of 11 leading to an extensive reduction in IFT values while using low salinity conditions is highly effective only for RSO sample oil. In this way, it seems that the presence of a high concentration of cations and anions along with the in-situ soap production (pH of 11) leading to a reduction in repulsive forces provides a better chance for the molecules to rearrange and accumulate in the interface causes a significant IFT reduction. The point is that a similar trend was reported by Sauerer et al.^68^ regarding the effect of high salinity conditions of NaCl (100,000 ppm), CaCl_2_ (100,000 ppm), and formation brine since they can reduce the IFT value to 5.3, 3.4, and 6.8 mN/m, respectively. They claimed that the reason for this is the stronger interaction of the monovalent sodium ion with the crude oil immanent surfactants, compared to the divalent calcium ion. Besides, Jha and his coworkers^69^ reported similar results for the lower value of IFT using sodium salts with different surfactants.

Also, Kakati, and Sangwai^70^ which used pure hydrocarbons instead of crude oil reported a better impact of NaCl solution than the CaCl_2_ solution for IFT reduction at the hydrocarbon-brine interface. They also revealed that it is possible to see both increasing and decreasing trends for the effect of salts on the IFT using the Jones–Ray effect^71–73^.

Furthermore, Lashkarbolooki et al.^74^ studied the influence of NaCl and CaCl_2_ on the IFT of acidic Iranian crude oil. According to the results reported by Lashkarbolooki et al., NaCl had a weaker effect on IFT reduction than CaCl_2_ which was correlated to the better interaction that existed between the immanent surface-active species of crude oil and divalent ions released due to the dissolution of CaCl_2_.

On the other side, there are several investigations dealing with the possible effect of crude oil composition on the IFT considering the different salts. In other words, it is well established that the relationship between IFT and salt concentration for multicomponent crude oil systems is rather complex, and might be difficult to find specific results and trends compared with pure hydrocarbon and water. For example, Buckley and Fan^75^ examined 41 crude oil samples from different origins around the world. They reported that the IFT of those oil samples were in the range of 3.0 to 27.3 mN/m using deionized water, 1.5–25.9 mN/m using an aqueous solution with NaCl concentration of 0.1 M sodium chloride (NaCl) and 2.4–26.9 mN/m using seawater. They reported that according to the measured IFT values, it is impossible to find a generalized correlation between IFT and the crude oil properties especially for complex brines such as synthetic seawater. Also, Okasha and Al-Shiwaish^76^ measured IFT for two different oil samples of dead and recombined crude oils under different salinities of 52, 108, and 215,000 ppm. They reported that for all of the examined concentrations, the effect of salinities was reduced on the measured IFT values while the temperature and pressure were held constant.

Furthermore, Isehunwa and Olubukola^77^ reported that the IFT reduces with increasing salinity, whereas Okasha and Al-Shiwaish^76^ observed IFT reduction as the formation brine was diluted. The point must be noticed is that the performed experiments by Isehunwa and Olubukola ^77^, and Okasha and Al-Shiwaish^76^ which used only NaCl and a complex formation brine, respectively, revealedthe crucial importance of brine composition on the IFT.

### Effect of ILs on the IFT reduction

In the next stage of this investigation, the effect of two ILs namely [C_10_mim][TfO] and [C_10_mim][Cl] were investigated on the IFT reduction by dissolving 500 ppm of these ILs in the aqueous solution. The measurements which are tabulated in Table 6 revealed that the presence of both ILs in the aqueous solutions regardless of the used sample oil led to a reducing effect on the IFT. A closer look into the results tabulated in Table 6 revealed that for the pH value of 11, the presence of ILs led to a significant reduction in IFT, especially for the [C_10_mim][TfO] leading to a minimum IFT value of 0.01 mN/m for the RSO/50,000 ppm Na_2_SO_4_.

**Table 6:** The effect of ILs on the IFT of the solutions under low and high salinity conditions.

	pH = 3.5		pH = 7		pH = 11	
	RSO	ASO	RSO	ASO	RSO	ASO
[C_10_mim][Cl] concentration = 500 ppm/Low salinity condition						
DW	16.6	14.4	9.7	10.5	0.21	1.1
Na_2_SO_4_	11.2	12.2	7.8	12.2	0.08	2.3
CaSO_4_	16.3	13.1	9.1	10.8	6.5	5.1
MgSO_4_	9.8	12.4	6.1	8.9	1.1	0.98
[C_10_mim][Cl] concentration = 500 ppm/High salinity condition						
DW	16.6	14.4	9.7	10.5	0.21	1.10
Na_2_SO_4_	10.2	8.4	5.7	8.1	0.06	0.76
MgSO_4_	7.1	9.3	9.8	5.1	0.95	0.44
[C_10_mim][TfO] concentration = 500 ppm/Low salinity condition						
DW	18.2	15.3	7.8	8.3	0.11	0.95
Na_2_SO_4_	15.4	16.1	6.4	5.2	0.05	0.55
CaSO_4_	17.1	12.3	7.6	9.8	4.30	3.82
MgSO_4_	16.1	14.4	5.1	6.7	0.88	0.31
[C_10_mim][TfO] concentration = 500 ppm/High salinity condition						
DW	18.2	15.3	7.8	8.3	0.11	0.95
Na_2_SO_4_	12.4	10.8	3.7	6.1	0.01	0.38
MgSO_4_	9.7	10.9	7.8	4.3	0.51	0.21

The reason behind this observed trend can be correlated to the higher efficiency of [C_10_mim][TfO] for IFT reduction than the [C_10_mim][Cl] and the higher acidic nature of the [C_10_mim][TfO] than [C10mim][Cl] comes from acidic nature of triflate part of the [C_10_mim][TfO]. In detail, as the results revealed, under an acidic pH condition of 3.5, the presence of [C_10_mim][TfO] led to higher IFT values compared with the aqueous solution modified by 500 ppm of [C_10_mim][Cl] since the triflate part of IL provides more acidic nature which is unfavorable in the presence of H + ions in acidic medium leading to repulsive forces in the interface. As a consequence of this repulsion, more repulsive forces existed in the solution moving the molecules to larger distances from each other to reduce the possible repulsive forces consequently leading to an increase in the IFT due to the lack of molecules in the interface. But, as the environment shifted toward neutral and basic conditions, the chance of reaction between the OH^−^ ions and the acidic compartment of the oleic phase and the acidic IL which here is [C_10_mim][TfO] enhances leading to the formation of in-situ soaps which can boost the effect of IL presence in the solution for IFT reduction. In this way, the minimum IFT was obtained for the solution comprised of 50,000 ppm of Na_2_SO_4_ + 500 ppm of [C_10_mim][TfO]/RSO under a pH value of 11. Moreover, a glance into the results tabulated in Table 4 revealed that RSO (TAN of 1.81) experienced IFT reduction higher than ASO (TAN of 0.45) for pH of 11 due to its higher acidic nature leading to a probable saponification process and in-situ surfactant production.

### Adsorption of ILs on the rock surface

In this phase of the investigation, 12 adsorption tests were performed using the optimum chemical formulations obtained for a pH value of 11 (due to the lowest IFT values obtained using this pH) no matter which IL was used in the first series of the adsorption test (see Table 7). These adsorption tests were performed using DW and saline solutions prepared with Na_2_SO_4_ with low and high concentrations since these aqueous solutions led to rather the lowest IFT value among the examined salts.

**Table 7:** Adsorption (mg IL/ g rock) of ILs on the rock surface as a function of salinity under pH value of 11.

	RSO	ASO
[C_10_mim][Cl] concentration = 1500 ppm/Low salinity condition		
DW	1.68	2.03
Na_2_SO_4_	1.78	1.86
[C_10_mim][Cl] concentration = 1500 ppm/High salinity condition		
DW	1.68	2.03
Na_2_SO_4_	1.89	1.63
[C_10_mim][ TfO] concentration = 1500 ppm/Low salinity condition		
DW	2.56	2.95
Na_2_SO_4_	2.24	2.48
[C_10_mim][ TfO] concentration = 1500 ppm/High salinity condition		
DW	2.56	2.95
Na_2_SO_4_	2.03	1.86

The performed adsorption tests showed that adsorption of both [C_10_mim][Cl] and [C_10_mim][TfO] for the system dealing with ASO is higher than the systems dealing with RSO with a maximum adsorption value of 2.03 and 2.95 mg IL/g rock, respectively. Based on these findings, the adsorption of [C_10_mim][Cl] with an adsorption value of 2.03 mg IL/ g rock is lower than the adsorption value obtained for [C_10_mim][TfO] with the value of 2.95 mg IL/g rock. The reason for this observed trend is directly correlated to the larger anionic section of [C_10_mim][TfO] compared with [C_10_mim][Cl]. As a consequence of this higher negative charge of the anionic group of [C_10_mim][TfO] with similar chain length ([C_10_mim]) to [C_10_mim][Cl], a stronger electrostatic binding between the positive surface of the carbonate rock and the IL molecules are formed consequently leading to higher adsorption of the [C_10_mim][TfO] molecules into the rock surface compared with the [C_10_mim][Cl]. Moreover, the results obtained for DW without IL showed that the RSO experienced lower adsorption than ASO on the rock surface. This observed trend is related to the higher negative surface charge of RSO because heteroatoms existed in their structure. The measured adsorption values revealed that the ILs experienced a lower adsorption value in the presence of ROS compared with ASO. The better performance of RSO for reducing the IL adsorption is related to its structure (lower aromaticity compared with the asphaltene). In detail, since the resin structure is more branched than the asphaltene, the head and tail resin structure acts as the natural surfactant and sacrifice for the IL. So, the binding between the negative surface of the resin molecules and the positive charge of carbonate rock reduces the chance of binding between the IL and carbonate rock. On the other hand, both used ILs are cationic surfactants with total positive charge which provide repulsive forces between the carbonate rock and IL molecules. So, this is the net effect of these two phenomena which strengthen each other and move the system to the point that a lower amount of ILs are adsorbed. The other point is that the adsorption of ILs on the rock surface in the presence of asphaltene is higher than the resinous synthetic oil due to the weaker negative surface charge of asphaltene. In detail, since the resins generally neutralize the charge of asphaltene surface charge for more stable particles in the solutions, it seems that the asphaltene molecules used to prepare the synthetic oil are more positive than the resin molecules extracted in the current investigation to prepare the resinous synthetic oil. As a consequence of these facts, the resinous synthetic oil is more successful in preventing the IL adsorption of the rock by sacrificing itself as the primary surfactant existing in the solution.

The other possible phenomenon that can manipulate IL adsorption is the formation of in-situ surfactants that mostly appear in systems dealing with alkaline and acidic oils. In this way, 4 IL adsorption tests under a pH value of 7 using DW were conducted since it can be assumed that there is no in-situ surfactant production under neutral condition (see Table 8).

**Table 8:** Adsorption (mg IL/ g rock) of ILs on the rock surface in the absence of salts and under pH value of 7.

	RSO	ASO
[C_10_mim][Cl] concentration = 1500 ppm condition		
DW	2.54	2.32
[C_10_mim][ TfO] concentration = 1500 ppm condition		
DW	3.46	3.12

The measured IL adsorption values revealed that for both ILs and all the examined synthetic oils, the adsorption was higher than those measured under a pH value of 11, especially for [C_10_mim][Cl] which faced more than a 50% increase in the IL adsorption. Since the only change in the performed adsorption tests is the pH, it is possible to correlate this higher adsorption to the absence of in-situ surfactant limitedly produced under a pH value of 7. As a consequence of this lower in-situ surfactant production, the available sacrifice agent for the rock surface reduces which means a higher chance for the IL molecules to be adsorbed on the rock surface.

In detail, the advantage of alkali injection for chemical EOR processes comes from its capability for in-situ soap production. As a consequence of this advantage, not only is the IFT reduced as the primary effect of this in-situ soap production, but also it is possible to control the surfactant adsorption by using the produced in-situ surfactants the sacrifice which directly affects the operating costs and significant improvement in the profit of EOR projects. Moreover, the application of alkali is desired since it can reduce the adsorption of surfactant on the rock surface as the pH is increased which consequently leads to higher surfactant stability^78^. But the point that must be carefully examined is that the impact of alkali in lowering the adsorption of surfactants is limited to situations where the salinity/hardness is in a reasonable range due to alkali sensitivity to divalent cations Ca^2+^ and Mg^2+^, which reduces its effectiveness and drives it to precipitate^79,80^.

For example, Somoza et al.^47^ investigated the adsorption of 4000 ppm [C_10_mim][TfO], 1 wt% NaOH, and 2 wt% NaCl on the Berea sandstone. They observed that there was no trace of IL in the effluent after 4 PVs of chemical formulation injection (4000 ppm IL, 1 wt% NaOH, 2 wt% NaCl) due to the high adsorption of the IL on the rock surface.

They reported this expected observed trend to the adsorption of ionic surfactants is strongly affected by the rock composition (with 4% of muscovite and albite) making the core surface negatively or positively charged. So it is unpreventable to see surfactant adsorption on the rock surface since the presence of negatively charged components of sand particles, such as silica, which are negative at neutral pH or in water can adsorb the surfactant molecules^58^. Besides, they performed another core flooding experiment using a carbonate core plug to investigate the possible adsorption of the IL on the carbonate rock. Their results revealed that the trace of IL was observed in the effluent just after 0.5 PV injection means the lower adsorption of the IL on the carbonate rock due to similar surface charge of both carbonate core plugs and surfactant molecules and possible interaction occurs between the calcite and other minerals containing Mg^2+^ and surfactant molecules^81^.

The point is that Somoza et al.^47^ reported similar results with the current investigation and the results reported by Nandwani et al.^82^ which correlated the obtained results to the repulsive force between the rock surface and surfactant which both of them are positively charged. As a consequence of this repulsion, lower surfactant adsorption especially for the imidazolium-based ILs than the pyrrolidinium or pyridinium-based ILs was obtained.

### Core flooding experiments

In the last stage of this investigation, two different core flooding experiments were performed using the optimum chemical formulation (50,000 ppm of Na_2_SO_4_ under a pH value of 11) using RSO as the sample oil (see Table 9). The core flooding experiments were designed and performed in a way that provides better information regarding the effect of used [C_10_mim][TfO] on the tertiary oil recovery. The main purposes of these core flooding experiments are a) to investigate the effect of chemical formulation on the tertiary oil recovery, and b) the synergy between the chemical formulation and used IL on the tertiary oil recovery. The obtained results tabulated in Table 6 revealed that using the optimum chemical formulation with the IL led to a maximum oil recovery of about 28.8% based on OOIP while the absence of IL in the optimum chemical formulation led to oil recovery of about 14.3% based on OOIP which means the significant effect of IL presence in the oil recovery.

**Table 9:** Effect of different chemical formulation on the tertiary oil recovery.

Run no	Properties of core plugs				Residual oil saturation after water flooding %	Water flood recovery %	Chemical floods			Chemical slug composition	Tertiary Recovery based on OOIP (%)
	Permeability (K) (mD)	Porosity ($\phi$) (%)	Length (cm)	Diameter (cm)			Slug Size pore volume (PV)	Injection Rate (cc/min)	pH	[C10mim][TfO] (ppm)	
1	10.2	15.5	8.1	3.78	43.5	47.3	0.3	0.3	11	0	14.3
2	13.3	13.6	8.3	3.78	45.9	44.6	0.3	0.3	11	500	28.8
3	11.8	16.9	8.1	3.78	44.8	43.9	0.3	0.3	7	500	10.1

before extracting any specific conclusion regarding the obtained results and the possible effect of IL and other chemicals that existed in the optimum formulation (50,000 ppm of Na_2_SO_4_ and RSO), another core flooding test was performed using neutral condition (pH of 7) and 500 ppm of [C_10_mim][TfO] dissolved in the aqueous solution which probably leading to no production of in-situ soap. The performed core flooding tests revealed that injection of prepared solution led to oil recovery of 10.1% based on the OOIP which means lower oil recovery than the situation that IL was used under basic conditions leading to about 14.5% higher oil recovery based on OOIP (saponification process). The first reason behind this observed trend is directly correlated to the adsorption of the IL in the absence of produced in-situ soap which reduces the ultimate effect of surfactant on the oil recovery since in the absence of in-situ soap production, surfactant molecules can be adsorbed on the rock surface. As a consequence of this probable adsorption, the effective concentration of IL reduces in the displacing solution leading to lower tertiary oil recovery.

In total, it seems that using the optimum chemical formulation has the potential to activate the IFT reduction mechanism leading to easier movement of trapped oil which is boosted by the probable wettability alteration that comes from the IL adsorption on the rock surface which can render the rock wettability from oil-wet condition to water-wet conditions. The point is that although the IFT reduction mechanism was observed, the impact of wettability alteration must be examined in detail using a complementary phase of this investigation. However, in the high pH conditions, the formation of in-situ soaps acts as the sacrifice and is adsorbed into the rock surface and even boosts the IFT reduction providing a better chance for the surfactant molecules to interact with the trapped oil drops and form a bigger oil bank leading to higher oil recovery efficiency in total. In general, surfactant adsorption in porous media is a fundamental and crucial concern in EOR processes which in severe cases surfactant loss owing to their interactions with reservoir rock and fluid directly reduces the effectiveness of the chemical solution injected, and may move the process toward uneconomical situation^58^.

In total, the researchers have reported that there are several effective parameters during the adsorption processes namely surfactant nature, solid/fluid system interaction, the electrostatic attraction between charged surface of the rock and charged head group of surfactant^24,32,78–80,83–85^ .

For example, Somoz et al.^47^ investigated the adsorption of 4000 ppm [C_10_mim][TfO], 1 wt% NaOH, and 2 wt% NaCl on the Berea sandstone. They observed that there was no trace of IL in the effluent after 4 PVs of chemical formulation injection (4000 ppm IL, 1 wt% NaOH, 2 wt% NaCl) which was an indication of high adsorption of the IL on the rock surface. They reported that the observed trend was expected and then correlated this trend to the fact that the adsorption of ionic surfactants is strongly influenced by the composition of the rock that makes the core surface negatively or positively charged. In their case which sandstone cores were used, the analysis revealed that the used core plugs are comprised of 4% muscovite and 4% albite which are silicate-like minerals while the used IL was a cationic surfactant. So it is unpreventable to see the adsorption of surfactant on the rock surface since the presence of negatively charged components of sand particles, such as silica, which are negative at neutral pH or in water can adsorb the surfactant molecules^58^. Besides, they performed another core flooding experiment using a carbonate core plug to investigate the possible adsorption of the IL on the carbonate rock.

Their results revealed that the trace of IL was observed in the effluent just after 0.5 PV injection which means the lower adsorption of the IL on the carbonate rock due to similar surface charge of both carbonate core plugs and surfactant molecules and possible interaction occurs between the calcite and other minerals containing Mg^2+^ and surfactant molecules^81^. The results reported by Somoz et al.^47^ and the obtained results in the current investigation are in good agreement with the results reported by Nandwani et al.^82^ regarding the effect of strong repulsion between positively charged rock surface and positively charged surfactant, prevents adsorption, especially for the imidazolium-based ILs than the pyrrolidinium or pyridinium based ILs.

As the last point, it seems that the higher oil recovery using the optimum chemical formulation is not only related to the lower IFT values of this formulation. The adsorption analysis showed inevitable adsorption of the ILs on the rock surface for the pH value of 11 and 7 although the adsorption for pH = 11 was lower than the adsorption value obtained for pH value of 7 (due to the formation of in-situ surfactant acts as the sacrifice for the IL surfactant). So, the wettability alteration due to IL adsorption is the other possible mechanism that enhanced the oil recovery due to surfactant injection. The point is that although the wettability alteration is the other possible mechanism, the portion of this effect is probably lower than the effect of IFT reduction because the wettability alteration is a time-consuming phenomenon while the IFT reduction instantly introduces its effect. For example, Uoda et al.^86^ reported that it is possible to reduce the contact angle of the rock surface from 98° to 28° using [C12Py][Cl] with concentration of 250 ppm.

Moreover, Sakthivel^87^ used Four imidazolium-based ILs, namely, 1-butyl-3-methylimidazolium chloride [C_4_mim]^+^[Cl]^−^, 1-hexyl-3-methylimidazolium chloride [C_6_mim]^+^[Cl]^−^, 1-octyl-3-methylimidazolium chloride [C_8_mim]^+^[Cl]^−^, and 1-decyl-3-methylimidazolium chloride [C_12_mim]^+^[Cl]^−^ to see if they can change the wettability of the rock surface. The results reported by Sakthivel^87^ revealed that using [C_12_mim]^+^[Cl]^−^ with concentrations of 0, 50, 100, 500, and 1000 ppm changed the contact angle to 82.5°, 62.2°, 54.9°, 49.4°, and 41.7°, respectively. In this way, it can be concluded that the ILs are applicable to change the wettability toward a desired condition of water-wet conditions.

In the last stage of this investigation, a comparison between the previously performed investigations on the tertiary oil recovery using ILs was performed for easier comparison between the efficiency of the optimum formulation obtained in the current investigation and the previously performed studies (see Table 10). A glance into the results tabulated in Table 10 revealed that in most of the examined crude oils, the heavy crude oils led to higher oil recovery due to the lower fingering effect and piston-wise movement of the formed oil bank compared with the light or medium crude oils provide better sweep efficiency into the core. A closer look into the results considering the crude oil density revealed that the higher tertiary oil recovery was obtained for the Omani crude oil with API° of 16 which is the lowest among the compared crude oils. As a consequence of these phenomena higher tertiary oil recovery can be produced through the tertiary stage. The other point that can be extracted through Table 10 is that according to the oil recovery values, the sandstone rocks with higher permeability have a higher potential to release higher amounts of trapped oils leading to higher overall tertiary oil recovery. The last point regarding the results given in Table 10 is that most of the solutions prepared with formation brine had more potential for higher tertiary oil recovery. The reason behind this observed trend can be correlated to the more complex nature of the formation brine than the simple solutions modified with salts. In detail, the presence of a diversity of ions in the solution has a higher impact on the oil recovery maybe by lowering the IFT value or changing the wettability of the rock surface since the surfactant molecules can provide more effective interactions with the different ions of the formation brine instead of a limited number of salts existed in the synthetic aqueous solutions. As a result of these interactions, higher oil recovery can be produced through the tertiary oil recovery processes.

**Table 10:** Comparison between the previously and current performed investigations for respect to the tertiary oil recovery^47^.

Surfactant	Aqueous solution	Oil type	Rock type	Rock permeability	Temperature	Recovery*
4000 ppm [C12mim]Cl ^40^	Formation brine	Iranian Crude Oil	Carbonate	µ = 8–16 mD	–	13
500 ppm Ammoeng 102^88^	20 wt% (83%NaCl, 17% CaCl2)	Saudi with API 28.37°	Berea Sandstone	µ = 300–450 mD	333.15 K	5
1.50 wt% Choline Chloride:Glycerol (1:2) ^89^	Formation brine	Omani Heavy Oil (API 16°)	Berea Sandstone	µ = 41.6 mD	353.15 K	29.8
2. 50 wt% Choline Chloride:Urea (1:2) ^89^	Formation brine	Omani Heavy Oil (API 16°)	Berea Sandstone	µ = 40.60 mD	333.15 K	30.8
3000 ppm PAAD + 5000 ppm [C_8_mim]Br ^90^	2 wt% NaCl	Simulated Crude Oil	Berea Sandstone	µ = 1500–600 mD	343.15 K	20.25
500 ppm L12:SDS (1:2.5) + 2000 ppm HPAM ^91^	Formation brine	Jidong Crude Oil	Sandstone	µ = 500–1000 mD	338.15 K	25
170 ppm [C_18_mim]Cl ^92^	Formation brine	Iranian with API 31.65°	Carbonate	µ = 15–30 mD	–	13
1000 ppm [C_12_mim]Cl ^93^	Seawater	Iranian with API 26°	–	µ = 1.8 mD	–	2.7
4000 ppm [P_4 4 4 14_]Cl + 5000 ppm NaOH ^94^	4 wt% NaCl	Model Oil WIOLTAN SHH 70	Berea Sandstone	µ = 300–1600 mD	@RT**	8.10
1500 ppm BMOT^95^	Formation brine	Shengli Crude Oil API 27.13°	Sandstone	µ = 470–515 mD	335.15 K	12.5
3500 ppm [C_18_mim]Cl ^46^	Formation brine	Iranian API 30°	Carbonate	µ = 3–8 mD	353.15 K	16.5
50,000 ppm of Na_2_SO_4_ under a pH value of 11 + [C_10_mim][TfO] (current investigation)	Distilled water modified with optimum formulation	Synthetic oil	Carbonate (outcrop)	µ = 10.2–13.3 mD	@RT**	28.8

*Tertiary oil recovery.

**Room temperature.

## Conclusions

The effects of sulfate-based salts of CaSO_4_, MgSO_4_, and Na_2_SO_4_ were examined on the IFT reduction, ionic liquid (IL) adsorption, and tertiary oil recovery. In this way, pH ranged between 3.5, 7, and 11 in the absence and presence of two ILs of 1-decyl-3-methyl imidazolium chloride ([C10mim][Cl]) and 1-decyl-3-methyl imidazolium triflate ([C_10_mim][TfO]) under the concentration of 500 ppm. The CaSO_4_, MgSO_4_, and Na_2_SO_4_ solutions with concentrations of 1500, 5000, and 5000 ppm, respectively, were prepared to mimic the low salinity conditions while 50,000 ppm of Na_2_SO_4_ and MgSO_4_ were used as the high salinity conditions. Besides, measured IFT values using resinous synthetic oil (RSO) and asphaltenic synthetic oil (ASO) (8 wt/wt % of resin and asphaltene) prepared by dissolving extracted resin and asphaltene based on the IP 143/90 method into toluene were examined. The obtained results revealed that:

- Increasing the pH value from 3.5 to 11 reduced the IFT of ASO/distilled water (DW) from 20.7 to 4.4 mN/m while for the RSO/DW IFT value was increased from 23.5 to 27.3 mN/m (pH = 3.5 → 7) in the first place and then reduced to 0.4 mN/m (pH = 7 → 11).
- The measured IFT values in this manuscript revealed better IFT reduction for pH enhancement from 3.5 and 7 if the salinity existed in the solution under high and low salinity conditions.
- Effect of salinity on the IFT reduction was more obvious under low salinity conditions for ASO since it has a neutral nature (TAN = 0.45 mg KOH/g) while for crude oil and RSO (TAN = 1.41 and 1.81 mg KOH/g oil) presence of salinity introduce no specific effect.
- The IFT significantly reduced as the pH was increased to 11 for all of the examined systems due to ionization leading to the generation of in-situ soap and saponification.
- Increasing the salinity to 50,000 for Na_2_SO_4_ leading to a significant reduction in IFT from 0.4 to 0.2 mN/m for RSO in the absence of ILs.
- The measurements revealed the increasing effect of high salinity conditions on the IFT for neutral conditions (pH = 7) and ASO while for the low salinity conditions, the trend was reducing.
- Dissolving 500 ppm of ILs namely [C_10_mim][Cl] and [C_10_mim][TfO] leading to a significant reduction in IFT especially, for the RSO under a pH value of 11 which means the possible synergy between the in-situ soap and used ILs as a new class of surfactants.
- The measurements revealed possibility of achieving ultra-low values of 0.01 mN/m using RSO synthetic oil/50,000 Na_2_SO_4_ + 500 ppm [C_10_mim][ TfO] with pH value of 11.
- The saponification process (increasing the pH from 7 to 11) led to a reduction the in IL adsorption for [C_10_mim][Cl] and [C_10_mim][TfO] from 3.67 mg IL/g rock to 2.33 mg IL/g rock and 4.21 mg IL/g rock to 3.34 mg IL/g rock respectively.
- RSO and ASO can retard the IL adsorption since they can be adsorbed on the rock surface as the natural surfactant in the preliminary stage of the adsorption process (acting as the sacrifice) consequently reducing the IL adsorption.
- The performed core flooding experiments using the optimum chemical formulation of RSO synthetic oil/50,000 Na_2_SO_4_ under a pH value of 11 revealed the efficiency of using the optimum chemical formulation for maximum oil recovery of 28.8% based on OOIP.

## Data Availability

All data generated or analyzed during this study are included in this published article.
